# Development of a Recombinase Polymerase Amplification Fluorescence Assay for the Detection of Canine Adenovirus 2

**DOI:** 10.3389/fvets.2021.596877

**Published:** 2021-12-20

**Authors:** Li Xiao, Mengdi Zhang, Zhige Tian, Ye Ge, Tongyuan Zhang, Li Yi, Feng Cong

**Affiliations:** ^1^Guangdong Laboratory Animals Monitoring Institute and Guangdong Provincial Key Laboratory of Laboratory Animals, Guangzhou, China; ^2^College of Coastal Agricultural Sciences, Guangdong Ocean University, Zhanjiang, China; ^3^Faculty of Agriculture, Forestry and Food Engineering, Yibin University, Yibin Key Laboratory of Zoological Diversity and Ecological Conservation, Yibin, China; ^4^Center for Animal Disease Control and Prevention of Fushun, Fushun, China; ^5^Institute of Special Economic Animal and Plant Science, Chinese Academy of Agricultural Sciences, Changchun, China

**Keywords:** limit of detection, fluorescence, real-time, canine adenovirus 2, recombinase polymerase amplification

## Abstract

Canine adenovirus type 2 (CAdV-2) is often found in co-infections with other pathogens causing canine infectious respiratory disease (CIRD). Rapid, efficient, and convenient pathogen detection is the best approach for early confirmatory diagnosis. In this study, we developed and evaluated a rapid real-time recombinase polymerase amplification (RPA) assay for detection of canine adenovirus 2 (CAV), which can detect CAV within 15 min at 39°C. The detection limit that assay was 214 copies/μl DNA molecules per reaction. The specificity was indicated by a lack of cross-reaction with canine distemper virus (CDV), canine coronavirus (CCV), and canine parvovirus (CPV). Field and clinical applicability of this assay were evaluated using 86 field samples. The coincidence rate of the detection results for clinical samples between CAV-RPA and qPCR was 97.7%. In summary, the real-time CAV-RPA analysis provides an efficient, rapid and sensitive detection method for CAV.

## Highlights

- CAV RPA has the shortest reaction time within 15 min among all the PCR-based methods.
- CAV RPA detection method has no cross-reactivity with CPV, CDV, and CCV.
- The sensitivity of CAV RPA was consistent with real-time PCR, as low as 214 copies/μl of DNA molecules per reaction.

## Introduction

Canine adenovirus (CAV) is a member of the genus *Mastadenovirus*, family Adenoviridae (1). Two distinct types of CAV, type 1 (CAV-1) and type 2 (CAV-2), are responsible for infectious canine hepatitis (ICH) and infectious tracheobronchitis (ITB), respectively (2). Canine adenovirus-type 1 strains have been reported worldwide from carnivore species included in the Canidae, Ursidae, and Mustelidae families (3). Canine adenovirus type-2 was first recovered in 1961 from dogs with laryngotracheitis (4), it is associated with mild infection of the respiratory tract and causes commonly widespread ITB (5). It has also been detected in the brains of dogs with neurological signs (6). With CAV-2, the route of infection is oronasal; it replicates efficiently in the respiratory tract and in the intestinal epithelium (7, 8). Therefore, efficient diagnosis is crucial in order to identify correctly the virus affecting a population of dogs.

Diagnosis of CAV infection was developed through virus isolation or nucleic acid-detection methods. Canine adenovirus induces the formation of cell clumps and Cowdry B intranuclear inclusion bodies after 48–96 h in cell culture (1). An RFLP assay was also developed for detection of CAV isolates. PCR and real-time PCR assays have also been developed for CAV detection (9–12). However, these methods are labor intensive and time-consuming (1).

The recombinase polymerase amplification (RPA) assay was developed as a novel isothermal method to amplify DNA efficiently under low-temperature conditions (between 37 and 42°C) (13). It utilizes a recombinase, DNA polymerase, and DNA-binding proteins to facilitate the insertion of oligonucleotide primers into their complement in a double-stranded DNA molecule (14).

In this study, we developed a rapid and sensitive RPA assay for detection of CAV-2, based on the E3 gene. It is sensitive, specific, and has the potential to be utilized as a point-of-care diagnostic tool in clinical diagnosis.

## Methods

### Ethics Approval and Consent to Participate

No animals were sacrificed specifically for this study. Fecal samples were collected by the veterinary hospital in Guangzhou, which is attended by owners of sick pets for diagnosis and treatment. If the veterinary hospital cannot confirm the pathogens associated with a disease, fecal samples are sent to Guangzhou Laboratory Animal Monitoring Institute for further pathogen diagnosis. In this process, we have no direct contact with pets. Finally, the diagnostic results are sent to the veterinary hospital.

### Viruses and Sample Collection

Canine parvovirus (CPV), canine distemper virus (CDV), and canine coronavirus (CCV) were cultured in MDCK cells, Vero cells, and CrFK cells, respectively, and stored in Guangdong laboratory animal monitoring institute. All of the cells were grown as a monolayer in Dulbecco's modified Eagle medium (DMEM) (GIBCO, Grand Island, NY, USA) and 5% CO_2_ in air. Eighty-six canine fecal swab samples were collected by the Guangdong laboratory animal monitoring institute. All the samples are processed in biosafety cabinets.

### DNA Extraction

Viral DNA was extracted using a DNA Mini Kit (50) (Omega Bio-tek, Norcross, GA, USA). The protocols in the instructions were followed. The genomic DNA was stored at −80°C.

### Generation of CAV DNA Standard

The sequence of the E3 gene is conserved within the whole CAV genome, therefore the E3 gene was chosen for preparing the DNA standard in this study. Viral genomic RNA and/or DNA were extracted either using TRIzol reagent (TaKaRa Biotechnology, Dalian, China) or using the DNA Mini Kit (50) (Omega Bio-tek, Norcross, GA, USA), following the manufacturer's protocol. The nucleic acids were stored at −80°C. A primer pair (E3-F: 5′-GAGTCTGCCCACGGGCCTATTT-3′; E3-R: 5′-CATGGACCTGATTTTGGTGTTT-3′) was used for the amplification of the E3 gene for ligation into the TA cloning vector pMD18-T. Positive standard plasmids of CAV were extracted using a Plasmid Mini Kit I (200) (Omega Bio-tek, Norcross, GA, USA), following the manufacturer's protocol.

### Primer and Probe Design

Canine adenovirus sequences based on the E3 gene were aligned using Lasergene software. One set of primers and probes based on the membrane gene of CAV (GenBank No. KY775393) were designed according to the recommendations from TwistDx Co., Ltd (https://www.twistdx.co.uk/en/support/rpa-assay-design-2). The primers were extended in the 5′ direction to 30 base pairs in length, according to the optimal amplification efficiency. A fluorescence-labeled exo-probe was designed according to the manual from TwistDx. All primer/probe sequences used in this study (forward primer: TAATTTCATTACCTTCAACATAACTGTACC; reverse primer: TTTAAACAGAGTCCATTCCATAAAACTGTC; probe: CAACTGGCAACAAAATCTAGTAGCCATATT[FAM-dT][THF]A[BHQ1-dT]CAACACGAGCCCCCAAAAATG) were synthesized by Sangon Biotech (Shanghai) Co., Ltd.

### Development of RPA Assays

Reagents for RPA were provided in RPA basic kits (ZC Bioscience, Hangzhou, China), and RPA reactions were performed according to the manufacturer's instructions. Briefly, a total of volume of 50 μl containing 2.5 μl of Mg^2+^ buffer (c = 280 mM), 300 nM of each primer, 120 nM probe, 10 ng DNA template, and sterile water were added and mixed with one tube of basic reaction unit.

The RPA reaction was carried out at 39°C in a Deaou-308C tube scanner (DEAOU Biotechnology, China). According to the manufacturer, a sample is confirmed positive if the amplification curve is above three and a half standard deviations (3.5 SD) of the background in the course of a valid time range (i.e., after 15–17 min of amplification). A threshold time range of 0–4 min and 30 s was used.

### Repeatability and Precision Variations of the RPA Assay

The standard plasmid was diluted to 10^5^ copies/μl, and was tested in three independent RPA reactions in 1 day or in 2 days. The coefficient of variation was analyzed using Graph prism 5.0.

### Analysis of Sensitivity and Specificity

In order to assess the sensitivity of the CAV-RPA, plasmid DNA containing the E3 gene sequence was serially diluted 10-fold (from 10^5^ copies/μl to 10^2^ copies/μl), and tested in three replicates for two independent runs to determine the detection sensitivity of the CAV-RPA assay. One microliters of each serially diluted plasmid was isothermally amplified using CAV-RPA primers at 39°C for 15 min. The threshold time was plotted against molecules detected and a semi-logarithmic regression was calculated using Prism 7.0 software (Graphpad Software Inc., California, USA). A probit regression was performed to determine the detection limit at 95% probability using SPSS Statistics software (IBM Corporation, New York, USA).

The specificity of the CAV-RPA assay was evaluated by testing a panel of viruses: CDV, CCV, and CPV. Genome extraction and RPA were performed as described above. A no template control served as the negative control.

### Testing of Field Samples

A total of 86 fecal swab samples were tested by the CAV-RPA assay and compared with qPCR to check any non-specific amplification (11). Ten percent (wt/vol) suspensions of the samples were prepared with DMEM. The supernatant fluids of the homogenates were harvested after passing through the 0.45 μm filter membrane. Total nucleic acids were extracted from fecal samples using the innuPREP MP basic kit A (Jena Analytic, Jena, Germany), following the manufacturer's instructions. The fecal samples were detected by RPA and qPCR, respectively.

## Results

### Sensitivity and Specificity of RPA

**Figure 1:**
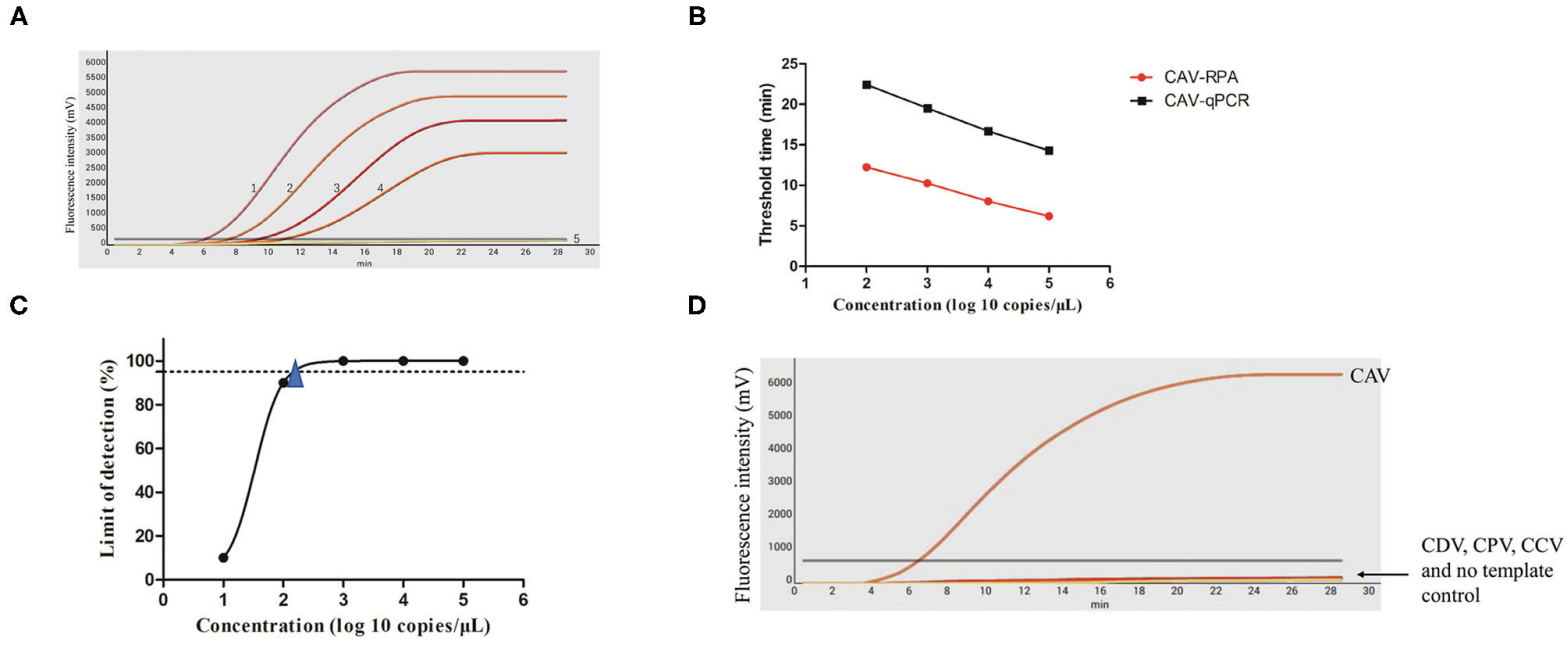
CAV-RPA assays. **(A)** Sensitivity of CAV-RPA assays was assessed with the 10^5^-10^2^ copies/μl DNA standard dilutions. 1: 10^5^ copies/μl, 2: 10^4^ copies/μl, 3: 10^3^ copies/μl, 4: 10^2^ copies/μl, 5: NTC indicates negative control. **(B)** Calibration curves of CAV-RPA assays (red dot *R*^2^ = 0.991) and CAV-qPCR assays (black square *R*^2^ = 0.995). Each dilution was tested using three replicates. **(C)** Analytical sensitivity of CAV-RPA assay (triangle). **(D)** Specificity of CAV-RPA assay was evaluated. Nucleic acids of CDV, CCV, and CPV were tested to assess specificity.

To evaluate the performance of the RPA amplification, we determined its sensitivity using serial dilutions of standard plasmid, which was 10-fold serially diluted ranging from 10^5^ to 10^2^ copies/μl. The sensitivity of CAV-RPA was 10^2^ copies/μl of target DNA, which is consistent with that of qPCR. Positive signals of 10^5^-10^2^ copies/μl were successfully detected within 6.0–12.0 min (Figure 1A). The detection limit, 214 copies per reaction at 95% probability, was further analyzed using 10 independent RPA tests (Figure 1C). Results from semi-logarithmic regression analysis showed a good correlation between molecule concentrations and detection time of the real-time RPA assay, with an *R*^2^-value of 0.991. Calibration curves of the CAV-RPA and qPCR showed that the detection time of CAV-RPA was shorter than that of qPCR (Figure 1B).

To test the specificity of the CAV-RPA assay, three other canine pathogens were used as templates. As shown in Figure 1D, positive products were amplified only from CAV, and the curves for CDV, CCV, and CPV showed no amplification. The specificity test of CAV-RPA revealed that the assay showed no cross-reactivity with the other canine viruses.

### Repeatability and Precision Variations of the RPA Assay

In order to verify the stability of the CAV-RPA assay, standard plasmids were diluted to 10^5^ copies/μl, and three independent RPA reactions in 1 day or in 2 days were carried out. The coefficient of variation was <5%, which showed that the CAV-RPA assay was repeatable and precise (Table 1).

**Table 1:** The day-to-day and within-day variations of the RPA assay.

**Within the day variation**			**Day-to-day variation**		
**TT**	**Mean**	**CV%**	**TT**	**Mean**	**CV%**
6:00	6:02	2.09%	5:90	6:04	2.3%
6:15			6:05		
5:90			6:18		

*Data listed in this table were from three independent RPA reactions using 10^5^ copies/μl of standard plasmid. CV, coefficient of variation; RPA, recombinase polymerase amplification; TT, threshold time*.

### Testing of Field Samples

A total of 86 rectal swabs from dogs with diarrhea, which were collected by the Guangdong Laboratory Animals Monitoring Institute, were tested for CAV using the RPA assay and compared with qPCR (11). Fifty of the 86 diarrhea samples were determined to be positive by the CAV-RPA. All the samples negative by CAV-RPA were negative by qPCR; 48 of the 50 CAV-positive samples determined by qPCR were positive, but the other two CAV-positive samples were negative. The amplicons of those two samples were purified and cloned into pMD18-T vector for sequencing. The result demonstrated that these two samples were positive for CAV. The coincidence rate of the detection results between qPCR and CAV-RPA was 97.7% (Table 2). To confirm the result of the CAV-RPA, amplicons of the 50 positive samples were obtained and sequenced. The generated sequences were 99% identical to that of the membrane gene of CAV.

**Table 2:** Coincidence rate of RPA and qPCR.

		**qPCR**			**CR**
		**Positive**	**Negative**	**Total**	
**RPA**	Positive	48	2	50	97.7%
	Negative	0	36	36	
	Total	48	38	86	

*CR, coincidence rate. CR = (48+36)/86 × 100%*.

## Discussion

Canine adenovirus is one of the viral agents implicated in the etiopathogenesis of ITB and has a high prevalence in canine populations (15).

Recombinase polymerase amplification is a rapid, sensitive, and convenient amplification technique (16). The reaction can be performed with minimal sample preparation, and the detection limit can be as low as 1–100 DNA target copies. The reaction can be performed in <15 min using simple isothermal amplification facilities and less well-trained staff (16). In this study, a CAV-RPA detection system based on the E3 gene, was developed, which demonstrates great advantages over existing detection methods. The detection time was reduced to <20 min, whereas the qPCR takes at least 1 h. In addition to saving time, the constant reaction temperature of 39°C allows the CAV-RPA assay to be performed using a simple lightweight portable device (ESEQuant tubescanner).

Moreover, the RPA assay can directly test original samples without nucleic acid purification, including blood, nasal swabs, and culture medium, and is conducive to spot detection (17). Many substances, nuclease-free water, heat lysis, alkaline lysis, PBS buffer, and DMEM, were used to dilute the samples for detection. In this study, DMEM was employed to dilute the fecal samples, and nucleic acids were obtained from the fecal samples using a magnetic bead-based kit. The results showed that the complex composition of the fecal samples and the fact that the samples may contain amplification inhibitors were tolerated by the RPA assay. Further experiments are planned to develop a set of portable RPA assays for use in remote regions for point-of-care diagnosis.

To summarize, we have developed a real-time RPA assay to amplify the E3 gene of CAV. It can detect CAV within 15 min at 39°C, and the detection limit of this CAV-RPA was 214 copies/μl. Furthermore, the CAV-RPA did not cross-react with CDV, CPV or CCV. Compared with qPCR, the RPA assay is more convenient and less time consuming. It provides the possibility of rapid clinical detection of CAV infection.

## Data Availability Statement

The raw data supporting the conclusions of this article will be made available by the authors, without undue reservation.

## Ethics Statement

The animal study was reviewed and approved by Animal Ethics Committee of Yibin University, Yibin, China, according to the OIE standards for use of animals in research and education. Samples were collected with permission from the farmer. Written informed consent was obtained from the owners for the participation of their animals in this study.

## Author Contributions

LX executed the study and helped to draft the manuscript. MZ, YG, TZ, and LY coordinated analysis of the data, and drafted the manuscript. LY, ZT, and FC conceived and designed the study, participated in the design of the study, and contributed to sample acquisition. All authors contributed to manuscript revision and have read and approved the submitted version.

## Funding

This work was supported by the Science and Technology Planning Project of Guangdong Province (2018B030317001), the Province prevention and control novel coronavirus infection of Guangdong Province (2020A111128021), Guangdong Science and Technology Comprehensive Affairs Management (163-2020-XMZC-0004-01-0009), the National Key Research and Development Program of China (No. 2021YFF0703300) and the Doctor Launch Project of Yibin University (Nos. 2019QD09 and 2019QD10), and the Jilin Province Scientific and Technological Program (20190301086NY).

## Conflict of Interest

The authors declare that the research was conducted in the absence of any commercial or financial relationships that could be construed as a potential conflict of interest.

## Publisher's Note

All claims expressed in this article are solely those of the authors and do not necessarily represent those of their affiliated organizations, or those of the publisher, the editors and the reviewers. Any product that may be evaluated in this article, or claim that may be made by its manufacturer, is not guaranteed or endorsed by the publisher.
